# Evaluation of In Vitro Antimicrobial, Antioxidant, and Anti-Quorum Sensing Activity of Edible Mushroom (*Agrocybe aegerita*)

**DOI:** 10.3390/foods12193562

**Published:** 2023-09-25

**Authors:** Aarti Bains, Prince Chawla, Baskaran Stephen Inbaraj

**Affiliations:** 1 Department of Microbiology, Lovely Professional University, Phagwara 144411, Punjab, India; 2 Department of Food Technology and Nutrition, Lovely Professional University, Phagwara 144411, Punjab, India; princefoodtech@gmail.com; 3 Department of Food Science, Fu Jen Catholic University, New Taipei City 242062, Taiwan

**Keywords:** *Agrocybe aegerita*, antioxidant activity, anti-quorum sensing activity, antibiofilm activity, HPLC, GC-MS, FTIR

## Abstract

In the present study, ethanol extract obtained from the mycelial culture of *Agrocybe aegerita* was evaluated for its antioxidant activity as well for its potential to inhibit the virulence factor responsible for quorum-sensing activity and antibiofilm activity of pathogenic *Pseudomonas aeruginosa* PAO1 strain. The extract of mushroom at different concentrations showed percentage inhibition in a dose-dependent manner for DPPH and nitric oxide assays with the lowest as 38.56 ± 0.11% and 38.87 ± 0.04% at 50 µg/mL and the highest as 85.63 ± 0.12% and 82.34 ± 0.12% at 200 µg/mL. FTIR analysis confirmed the presence of functional group -OH, O-H bending bonds, C=C stretching, pyranose ring, and H-C-H stretch, confirming the presence of phenol, carotenoid, and ascorbic acid. HPLC analysis revealed that the concentration of gallic acid present in the extract is 27.94 mg/100 g which is significantly (*p* < 0.05) more than the concentration of rutin (i.e., 7.35 mg/100 g). GC-MS analysis revealed the presence of 5-methyl-1-heptanol, 2-heptadecenal, phthalic acid, butyl hept-4-yl ester, 2-dodecanol, benzoic acid, TMS derivative. The extract showed significantly (*p* < 0.05) more inhibition of pyocyanin (61.32%) and pyoverdine (54.02%). At higher concentrations of mushroom extract, there was a significant (*p* < 0.05) reduction (56.32%) in the swarming motility of the test organism. The extract showed 72.35% inhibition in biofilm formation. Therefore, it has been concluded from the present study that mushroom extract, which is rich in phenolic compounds interferes with the virulence factor responsible for quorum sensing, thereby inhibiting biofilm formation, and can be utilized as therapeutic agents against multi-drug resistant pathogenic microorganisms.

## 1. Introduction

Mushrooms have gained popularity as a functional food and as a source for the development of pharmaceutical and nutraceuticals [1]. The fleshy and palatable fruit bodies of several species of macro-fungi are termed edible mushrooms. Perhaps edibility is evaluated by criteria that are based on factors such as the absence of human-toxic effects and pleasant aroma and taste [2]. Humans consume edible mushrooms for their nutritional value as comestibles and sometimes for their reported potential health benefits [3]. Edible mushrooms are not acknowledged as therapeutics or medical treatments since mushrooms have not yet been validated to treat medical conditions by mainstream science or medicine [4]. Some edible mushroom isolates have indeed been found to have preliminary evidence for cardiovascular, anticancer, antiviral, antibacterial, antiparasitic, anti-inflammatory, and antidiabetic effects [5]. Several extracts are currently widely used in Japan, Korea, and China as potential adjuvants to chemotherapy and radiation treatments. Mushrooms are considered to have substantial medicinal benefits, comprising anticancer, antimicrobial, antiviral, immune response-stimulating, and blood lipid-lowering qualities [6]. Different mushrooms’ fruiting bodies and mycelial culture both encompass multiple compounds, including terpenoids, steroids, polyphenols, polyketides, poly glucan, flavonoids, alkaloids, polysaccharides, and dietary fibers that have a diversity of pharmacological properties; therefore, they are considered as a rich source of antibiotics, antineoplastics, and antioxidants. Among edible mushrooms, *Agrocybe aegerita* (Brig.) a white rot basidiomycete termed Sing is commercially cultivated in different parts of the world [7]. Due to its frequent occurrence on poplar wood logs, the mushroom is also known as the “black poplar mushroom” or “chestnut mushroom”. It can be found throughout Asia, Europe, and North America. The protein-rich content in *Agrocybe aegerita* (25–30% of its weight) provides humans with exquisite nutrients [8]. Importantly, the mushroom has been used for hundreds of years by Asian people, notably the Chinese, as a herbal medicine to promote diuresis and improve spleen functions that are related to hyperuricemia [9]. In addition, in the recent era, the rise in antibiotic resistance among microorganisms is a significant global health concern and has outpaced the development of new antibiotics. The resistance of microorganisms toward antibiotics and the host immune system is due to their ability to form biofilm, a complex structure that adheres to the surface, and secrete a protective matrix [10]. Another factor that is responsible for the resistance of bacteria toward antibiotics is quorum sensing, a mechanism that is used by bacteria to communicate with each other through the release and detection of autoinducers known as signaling molecules [11]. These autoinducers accumulate when the bacterial population reaches a certain threshold and bacteria can sense their presence. This enables them to coordinate their behavior as a group [12]. Therefore, to combat drug-resistant bacterial infections, new therapeutic measures have been developed and it has been reported that the genus *Agrocybe aegerita* comprises a range of bioactive metabolites, notable polysaccharides with hypoglycemic potential indole derivatives with free radical-scavenging activity, and agrocybin, a peptide with antimicrobial potential [13]. Due to biological activities such as antioxidant activity, antimicrobial activity, anti-inflammatory as well immune-modulatory, mushroom polysaccharide has received increasing attention in recent years. Numerous techniques depending on multiple concepts have been developed for polysaccharide extraction. For instance, polysaccharides have been extracted by hot water extraction, hot alkaline extraction, ultrasound-assisted extraction, microwave-assisted extraction, superheated water extraction, and enzymatic method extraction [14,15]. Owing to their prolonged durations, high temperatures, low efficiency, and risk of polysaccharide degradation, these conventional extraction methods require significant amounts of energy and time [15]. Therefore, the present study aims to prepare ethanol extract from the mycelial culture of *A. aegerita* using a modified evaporated technique. An analysis of the extract was performed to identify functional groups of phenolic compounds as well as the amount of phenols and flavonoids present in them using FTIR, HPLC, and GC-MS techniques. Furthermore, the extract was studied to evaluate its antioxidant, anti-quorum sensing, and antibiofilm activities. Overall, the study represents a significant contribution of edible mushrooms in curing diseases caused by multidrug-resistant microorganisms by inhibiting the virulence factor responsible for quorum sensing and biofilm formation; therefore, mushrooms can have a significant contribution in pharmaceutical as well as food industries.

## 2. Materials and Methods

### 2.1. Materials

The mycelial culture of *A. aegerita* was obtained from the Directorate of Mushroom Center, Solan, Himachal Pradesh, India. The analytic reagent that includes FCR (Folin–Ciocalteu reagent), PBS (phosphate buffer), NaCl (sodium chloride), dimethyl sulfoxide (DMSO), ascorbic acid, 2-[(2,6-dichlorophenyl)amino]benzeneacetic acid sodium salt, aluminum chloride, sodium carbonate, metaphosphoric acid, DPPH (2,6-dichlorophenol, 2,2-diphenylpicrylhydrazyl), *a*-naphthyl ethylenediamine, sulphanilic acid, phosphoric acid, ethanol, and media Mueller Hinton agar, ME broth (malt extract) were obtained from Hi-Media Limited, Mumbai, India. Standard gallic acid, rutin, and other chemicals like methanol, acetonitrile, and HPLC-grade water were obtained from Sigma Aldrich, St. Louis, MO, USA. Deionized water and glassware rinsed with 10% nitric acid were utilized to carry out research experiments. The potential of the mushroom extract against biofilm formation and quorum sensing was studied using the *Pseudomonas aeruginosa* PAO1 strain.

### 2.2. Extract Preparation

The extract preparation from the mycelial culture of *Agrocybe aegerita* was performed by a modified solvent evaporation method outlined by Shashikant et al. [14]. Briefly, the mushroom mycelial culture was cultured in an Erlenmeyer flask (250 mL) that contained malt extract broth. The sample was incubated at 27 °C for fifteen days. The mycelial culture was then filtered using Whatman filter paper 1 and dried at 32 °C for 72 h. The sample was ground to a fine powder using a mechanical grinder (Glen, 350 W, 20,000 RPM mixer, grinder, blender, Dublin, Ireland). The powdered sample (5 g) was then mixed with 50 mL of absolute ethanol and a conical flask containing the mixture was kept in shaking condition in an orbital shaker (Thermo Scientific, Mumbai, India) at 500 rpm for 48 h. The supernatant was filtered using Whatman grade 4 paper. The supernatant was then kept at 4–7 °C in a refrigerator to evaporate the solvent ethanol. The milky white viscous extract thus obtained was kept in vials and stored at −20 °C for further analysis.

### 2.3. Characterization

#### 2.3.1. Determination of Functional Group and Quantification of Phenolic Compounds

The functional group present in the ethanol extract of mycelial culture was determined by the method followed by Bekiaris et al. [15] using FTIR (Fourier Transform Infrared) spectroscopy (Bruker FTIR Alpha spectrometer). Briefly, the mycelial sample (4 mg) was kept on the machine lens mirror surface and the lens was adjusted to obtain spectra in the mid-infrared scanning range of 4000–600 cm^−1^.

The quantification of phenolic compounds, namely gallic acid and rutin, present in *A. aegerita* extract, was performed by the method followed by Bristy et al. [16] using high-pressure liquid chromatography (Arc HPLC System, Waters India Pvt. Ltd., Bengaluru, Karnatka). The system consists of a temperature-controlled column chamber, gradient binary pump system, injector (manual), and photodiode array detector (2998) for the quantification and detection of phenol and flavonoids. Empower2 software was used to collect and analyze the obtained data. The C18 column 4.6 × 250 mm, 33 cm (Waters) with a 60 mL/h flow rate was used for rutin and gallic acid analysis. The rutin and gallic acid were quantified at 357 nm and 272 nm, respectively.

#### 2.3.2. Identification of Bioactive Compounds Using GC-MS Technique

The identification of bioactive compounds present in *A. aegerita* was carried out by Gas chromatography-mass spectrometry (Thermo Fisher Scientific, Waltham, MA, USA).The instrument was set with GC 1300 gas chromatography, autosampler (TriPlus RSH), TSQ Duo Mass selective quadrupole detector, and Trace Gold TG5MS column (40 m length, internal diameter 0.15 mm, and film thickness 0.15 m). The compounds present in the extract were diluted in n-hexane (1:99) and the initial temperature was kept at 62 °C to 185 °C for 60 to 180 s, final temperature was kept at 235 °C for 15 min, and in splitless mode, 1 µL of sample (10 µg/mL) was injected with autosampler. The complete analysis was kept for 35 min by maintaining a constant temperature of 10 °C/min. The program was run at the rate of 0.6 mL/min with linear velocity of the helium carrier gas. The electron impact was kept at 65 eV and used as an ion source temperature with 245 °C of transfer line temperature at 225 °C. The sector of the mass analyzer was set to scan from 45 to 450 *m*/*z*. Gas chromatographic and mass spectrometric data were processed by using Xcalibur software (Version 10).

### 2.4. Antioxidant Assay

#### 2.4.1. DPPH Free Radical Scavenging Assay

The estimation of the antioxidant activity of the ethanolic extract of *A. aegerita* was performed by the method given by Baliyan et al. [17]. Herein, extract (250 µL) was added to the DPPH solution (2.5 mL, 0.1 mM) and test tubes were incubated in a dark place for 30 min. The absorbance of the resultant mixture was observed using a UV spectrophotometer (Shimadzu, UV 3600i, Kyoto, Japan). The potential of mushroom extract to scavenge DPPH was determined by the following Equation (1):

(1) $$\%\;\mathrm{inhibition}=\frac{\mathrm{Absorbance}\;\mathrm{of}\;\mathrm{control}-\mathrm{Absorbance}\;\mathrm{of}\;\mathrm{reaction}\;\mathrm{mixture}}{\mathrm{Absorbance}\;\mathrm{of}\;\mathrm{control}}\times 100$$

#### 2.4.2. Nitric Oxide Assay

The interaction of the *A. aegerita* mushroom extract with nitric oxide was determined by the nitrite detection method followed by Adebayo et al. [18]. In brief, sodium nitroprusside (10 mM) dispersed in PBS (0.5 M) was used as the chemical source of nitric oxide. The mixture was dissolved properly and kept at constant condition for 5 h at room temperature. To the mixture, 400 µL Griess reagent (sulphanilic acid 1% in 5% H_3_PO_4_ and α-naphthyl ethylenediamine 0.1% in water) was added and absorbance was taken at 546 nm. The potential of mushroom extract to scavenge nitric oxide was determined by following Equation (2)

(2) $$\%\;\mathrm{inhibition}=\left[\left(\frac{\mathrm{Absorbance}\;\mathrm{of}\;\mathrm{control}-\mathrm{Absorbance}\;\mathrm{of}\;\mathrm{reaction}\;\mathrm{mixture}}{\mathrm{Absorbance}\;\mathrm{of}\;\mathrm{control}}\right)\times 100\right]$$

### 2.5. Determination of Virulence Factor in P. aeruginosa Responsible for Quorum Sensing

#### 2.5.1. Estimation of Pyocyanin Production

The estimation of pyocyanin production was performed by the method followed by Rodriguez-Urretavizcaya et al. [19] with slight modification. In brief, for the quantification of the production of pigment pyocyanin, the microorganism was inoculated in PB (Pseudomonas broth) medium (MgCl_2_ 1.4 g/L, K_2_SO_4_ 10 g/L, and peptone 20 g/L). The test organism was taken in four separate test tubes containingfour different concentrations of mushroom extract (200, 400, 600, 800, and 1000 µg/mL) and a test tube without extract was considered as a control. All the test tubes were kept for incubation at 37 °C in an orbital shaker incubator for 12–18 h. The test-treated and untreated test cultures were centrifuged (Lab Stac CEN 17-16R table top refrigerated centrifuge) at 15,000 rpm for 8 min. The supernatant (4 mL) obtained is dispersed with chloroform (2 mL) followed by the addition of HCl (500 µL of 0.2 M) in the organic phase. The absorbance of the deep red/pink color layer was taken at 520 nm using a UV-spectrophotometer (Shimadzu, UV 3600i, Kyoto, Japan). The pyocyanin content (µg/mL) was calculated by following Equation (3).

(3) $$\mathrm{Pyocyanin}\left(µg/mL\right)=\left[\left(\mathrm{Optical}\;\mathrm{density}\;\mathrm{at}\;520\;\mathrm{nm}\times 17.072\right)\right]$$

#### 2.5.2. Estimation of Pyoverdine Production

The pyoverdine estimation was performed by the method followed by Shukla et al. [20]. Herein, the supernatants containing different concentrations (200, 400, 600, 800, 1000 µg/mL) of extract and test microorganisms and of positive control were collected. The supernatant (200 µL) was then treated with 800 µL Tris HCl (50 mM, pH 7.4). The mixture showed yellow-green fluorescence at 405 and 465 nm (excitation and emission wavelength) when observed under a spectrofluorometer (Shimadzu, Japan). A relative fluorescence unit was used to express the activity.

#### 2.5.3. Swarming Motility

The QS inhibitory effect of the mushroom extract on swarming motility in *P. aeruginosa* was performed by the method described by Hou et al. [21]. In brief, Luria agar (LA 0.5%) plates containing different concentrations of mushroom extract (200, 400, 600, 800, 1000 µg/mL) were prepared. Bacterial cultures (10 µL) were inoculated in the center of LA plates. A plate containing no extract was taken as a positive control. All the plates were kept for incubation at 37 °C for 18 h, and after incubation, the swarm zones were obtained in the plates containing different concentrations of extract and were measured in mm from the point of inoculation.

### 2.6. Antibiofilm Activity

The antibiofilm activity of *A. aegerita* extract was determined by the ELISA plate method followed by Bouaouina et al. [22] with certain modifications. Briefly, *P. aeruginosa* PAO1 culture (50 µL) was added in the first well, followed by the addition of extract (100 µL), and then dilution was made up to the 11th well, while the 12th well was taken as control (no addition extract). The microtiter plates were incubated at 37 °C for 72 h. The culture was then removed from each well and wells were rinsed with 0.9% NaCl and dried for 15 min at 60 °C followed by the addition of crystal violet and incubation at 27 °C for 15 min. The crystal violet retained by cells was obtained by rinsing the wells with 0.9% NaCl and 30% acetic acid. Absorbance was observed at 595 nm using an ELISA plate reader. The percentage inhibition was calculated by following Equation (4).

(4) $$\mathrm{inhibition}\left(\%\right)=\left[1-\left(\frac{\mathrm{The}\;\mathrm{absorbance}\mathrm{of}\mathrm{the}\mathrm{test}\mathrm{sample}}{\mathrm{Absorbance}\mathrm{of}\mathrm{control}}\right)\times 100\right]$$

### 2.7. Statistical Analysis

The experiments performed in the present study were carried out three times and the data obtained was represented as the mean of the three independent determinations ± standard deviation. The obtained data were subjected to a t-test and one-way analysis of variance (ANOVA) to calculate the statistically different mean values at *p* < 0.05 [23]. Microsoft^®^ Excel, 2021 (Microsoft Corporation, Redmond, WA, USA) was used to compare mean values estimated by the critical difference (CD) value.

## 3. Results and Discussion

### 3.1. Characterization and Estimation of Bioactive Compounds of Agrocybe aegerita

**Figure 1:**
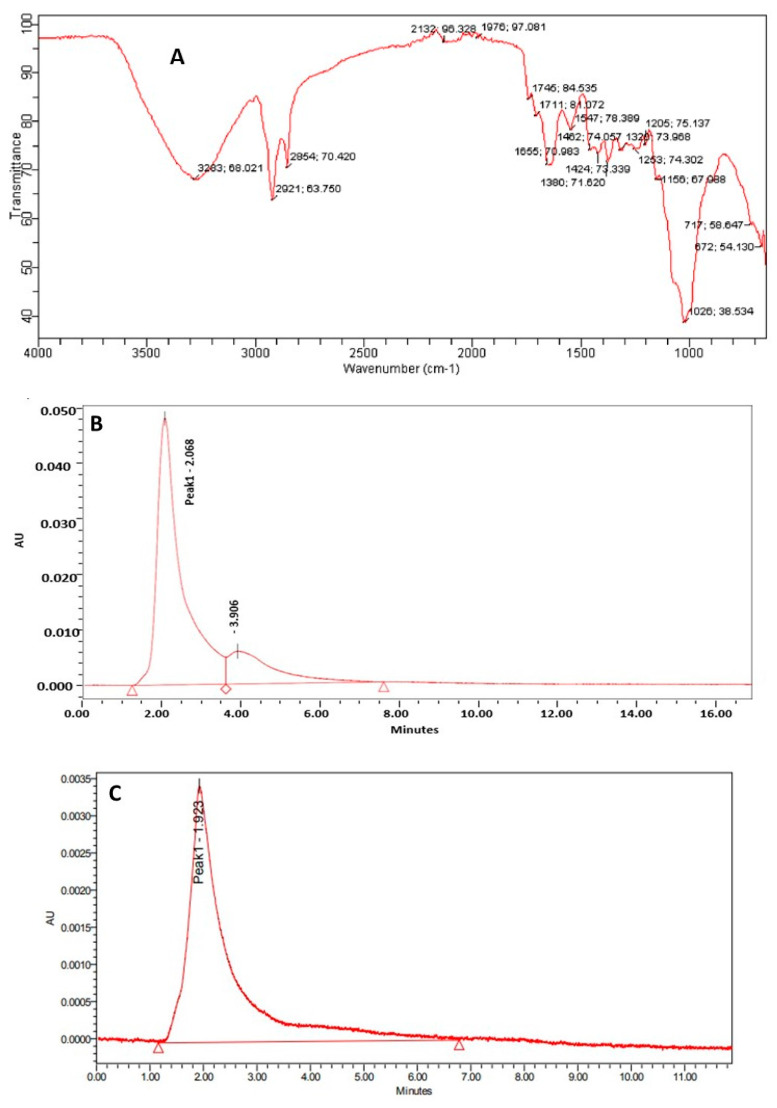

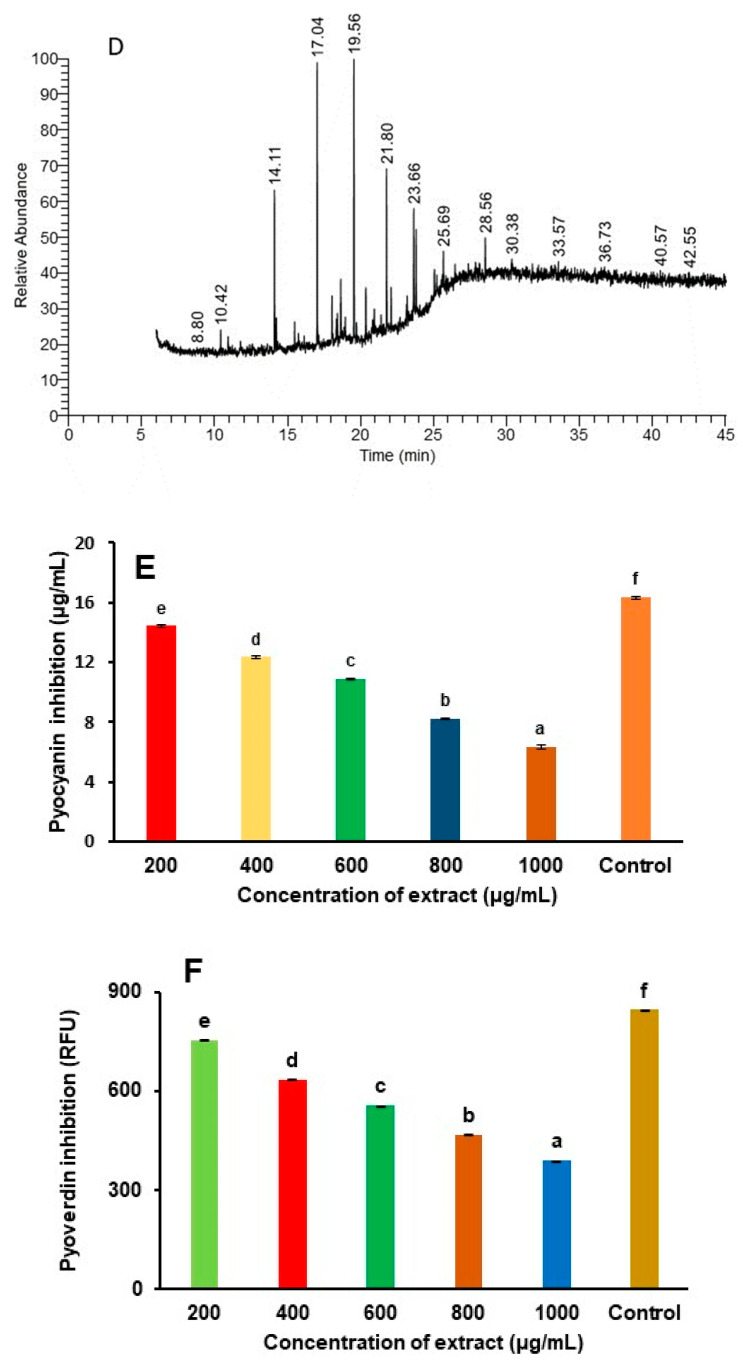

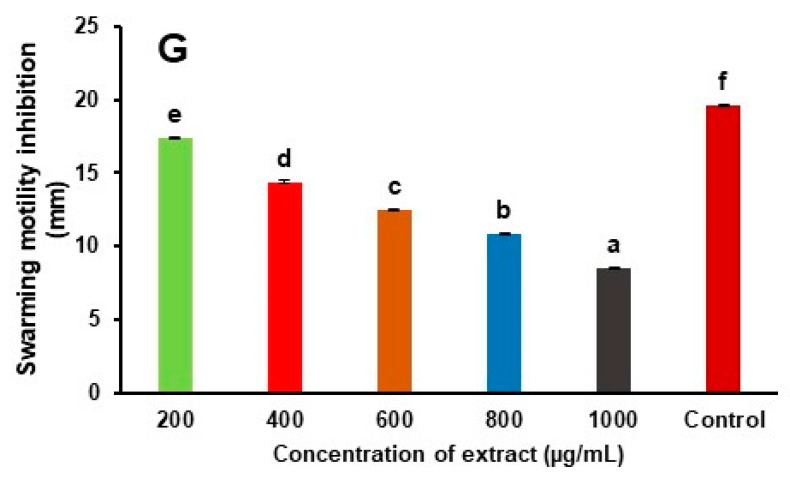
(**A**) FTIR spectra of ethanol extract of *A. aegerita*; (**B**) HPLC chromatogram of ethanol extract of *A. aegerita* for estimation of gallic acid; (**C**) HPLC chromatogram of ethanol extract of *A. aegerita* for estimation of rutin; (**D**) GC-MS chromatogram of bioactive compounds isolated from methanol extract of *A. aegerita*; (**E**) Effect of ethanol extract of *A. aegerita* upon inhibition of virulence factor pyocyanin responsible for quorum sensing. The results were expressed as mean ± standard deviation of >3 independent replicates and error bars represent the standard deviation from mean values, while different lowercase letters (a–f) above each bar represent significant differences (*p* < 0.05) from each other; (**F**) Effect of ethanol extract of *A. aegerita* upon inhibition of virulence factor pyoverdine responsible for quorum sensing. The results were expressed as mean ± standard deviation of >3 independent replicates and error bars represent the standard deviation from mean values, while different lowercase letters (a–f) above each bar represent significant differences (*p* < 0.05) from each other; (**G**) Effect of ethanol extract of *A. aegerita* upon inhibition of virulence factor Swarming motility responsible for quorum sensing. The results were expressed as mean ± standard deviation of >3 independent replicates and error bars represent the standard deviation from mean values, while different lowercase letters (a–f) above each bar represent significant differences (*p* < 0.05) from each other.

The extract obtained after evaporation was viscous and pale yellow, and the total yield of the extract was 0.25 mg/250 mL. The functional group of phenolic compounds is represented in Figure 1A. Herein, at 3283 cm^−1^ we confirm the presence of the −OH group. The peaks at 2921 cm^−1^ and 1380 cm^−1^ were due to O−H bending bonds, and the peak at 1655 cm^−1^ represents C=C stretching that is due to the conjugate double bond and represents the presence of carotenoid. The peak at 1742 cm^−1^ was due to C=O stretching vibration and represents the presence of ascorbic acid [24]. The peak at 2858 cm^−1^ is due to the presence of a pyranose ring, and stretching at 1547 cm^−1^ is due to the symmetric or asymmetric H−C−H stretch that indicates the availability of alkanes [25]. At 1156 cm^−1^ there is a vibrational band that is due to the glycosidic bonds [26]. Furthermore, the estimation of phenolic compounds, mainly gallic acid and rutin, was achieved by the HPLC method and results are represented in Figure 1B,C. The chromatogram revealed that the extract showed a sharp peak at a retention time of 2.068 min and 1.923 for gallic acid and rutin, respectively. The analysis showed that the concentration of gallic acid present in the extract is 27.94 mg/100 g which is significantly (*p* < 0.05) more than the concentration of rutin (i.e., 7.35 mg/100 g). The gallic acid and rutin observed have significant pharmacological applications such as antimicrobial, antioxidant, and anti-inflammatory activities and, therefore, can be utilized to cure different diseases. The present study aligns with the study by Bristy et al. [16] who isolated phenolic compounds from three different species of mushrooms: *Calocybe indica*, *Ganoderma lucidum*, and *Ganoderma tropicum*.

### 3.2. Identification of Mushroom Bioactive Compounds Using GC-MS Analysis

The identification of bioactive compounds isolated from the mycelial culture of *A. aegerita* was carried out using the gas chromatography-mass spectroscopy method and results are represented in Figure 1D and Table 1. The peaks were recorded at retention times 10.42, 14.11, 17.04, 19.56, 21.80, 23.66, 25.69, 28.56, 30.38, 33.57, 36.73, 40.57, and 42.55, and revealed the presence of 5-methyl-1-heptanol, 4-nonene, 3-methyl-, (Z)-, 1-hexadecanol, 2-heptadecenal, 10-heneicosene (c,t), phthalic acid, butyl hept-4-yl ester, 2-dodecanol, 9H-carbazole-1-carboxylic acid, 4-(1H-indol-3-yl)-, methyl ester, 2-dodecanol, 9H-carbazole-1-carboxylic acid, 4-(1H-indol-3-yl)-, methyl ester, ethanol, 2-(methylamino)-, benzoic acid, TMS derivative, acetamide, 2,2,2-trifluoro, 7-hydroxy-7,8,9,10-tetramethyl-7, 8-dihydrocyclohepta[d,e] naphthale, and pyrazolo [1,5-a]pyridine, 3-methyl-2-phenyl. The present results are in accordance with the findings of Li et al. [27] who evaluated the nutritional and health properties of *Agrocybe striatipes.*

### 3.3. Antioxidant Activity

The antioxidant potential of the ethanol extract of *A. aegerita* is represented in Table 2 and Figure 1B,C. Herein, in comparison to the extract, the control ascorbic acid showed significantly higher (*p* < 0.05) percentage inhibition for both assays. The ethanol extract of mushroom showed percentage inhibition in a dose-dependent manner with lowest 38.56 ± 0.11, 38.87 ± 0.04% at 50 µg/mL and highest 85.63 ± 0.12 and 82.34 ± 0.12% at 200 µg/mL for DPPH and nitric oxide scavenging assays. Therefore, it has been reported from the present study that bioactive compounds, namely phenol and flavonoids, present in mushroom extract allowed it to donate hydrogen to DPPH radicals, converting it into yellow-colored diphenylpicrylhydrazine which indicates the reduction of radicals [28]. Furthermore, in the case of nitric oxide scavenging assay, sodium nitroprusside when dissolved in an aqueous solution at physiological pH (approximately 7.4) spontaneously releases nitric oxide (NO) molecules that react with oxygen to form nitrogen dioxide which is further converted to nitrite ions in the presence of water. The generated nitrite ions were quantified using Griess reagent, which results in the formation of pink azo dye. The mushroom extract, due to the presence of bioactive compounds, scavenged the nitric oxide for oxygen, thereby reducing the production of nitrite ions [29]. The present results align with the study of Sifat et al. [30] and Stojanova et al. [31], who evaluated the antioxidant effect of ethanolic extract of *Auricularia auricular*, *Ganoderma lucidum*, *Pleurotus citrinopleatus*, *Pleurotus djamor*, *Pleurotus eryngii*, *Pleurotus ostreatus*, *Pleurotus ostreatus, Suillus granulatus*, *Coriolus versicolor*, and *Fuscoporia torulosa*.

### 3.4. Reduction of Quorum Sensing Linked Virulence Factor

The different concentrations of the mushroom extract were evaluated to study the reduction of virulence factors responsible for quorum sensing in *P. aeruginosa* PAO1, which includes pyocyanin, pyoverdine, and swarming ability. There was a significant decrease in the production of pyocyanin and pyoverdine in the presence of different concentrations of mushroom extract. The mushroom extract showed significantly (*p* < 0.05) more inhibition of pyocyanin (61.32%) and pyoverdine (54.02%) at a concentration of 1000 µg/mL and the least inhibition of pyocyanin (11.68%) and pyoverdine (10.78%) at a concentration of 200 µg/mL. Furthermore, the swarming motility of the test organism was evaluated in LA medium and it has been observed that at higher concentrations of mushroom extract, there was a significant (*p* < 0.05) reduction (56.32%) in the swarming motility of the test organism. The results are shown in Table 3 and Figure 1E–G. The pyocyanin, due to its redox-active properties, results in the interference of various cellular functions thereby promoting the pathogenicity of *P. aeruginosa.* Moreover, on solid surfaces for the development of biofilm, it also plays a secondary role [32]. Pyoverdine is another major virulence factor that is released by lipocalin, a neutrophil gelatinase that promotes severe infection in case of cystic fibrosis. In addition to this, pyoverdine, a siderophore, initiates a surveillance pathway and disrupts different biological functions by chelating iron from host iron sequester factors [33]. In the present study, mushroom extract inhibits these virulence factors in a dose-dependent manner. The reduction of pyocyanin and pyoverdine could be due to the presence of phytocompounds such as 5-methyl-1-heptanol, 2-heptadecenal, phthalic acid, butyl hept-4-yl ester, 2-dodecanol, benzoic acid, TMS derivative as well as phenol and flavonoids isolated from mushroom extract by GC-MS and HPLC analysis. Moreover, it has been reported in various studies that in *P. aeruginosa* PA01 the expression of quorum-sensing genes such as lasR, lasI, lasA, lasB, rhlA, rhlI, rhlR, phzA1 are reduced by the phenolic compounds, mainly flavonoids [34]. Pyocyanin can reduce oxygen molecules, thereby promoting redox reaction that results in cell death. The polyphenols and flavonoids reported in the present study are known to have antioxidant properties and could result in the reduction of oxidative stress due to the redox reaction caused by pyocyanin [35]. Furthermore, the reduction of the swarming activity of *P. aeruginosa* PA01 may also be due to the presence of phenolic compounds, namely gallic acid and rutin, in the mushroom extract that target the quorum-sensing pathway or receptor itself leading to a disruption of the quorum-sensing system. The present results align with the findings of Krishanan et al. [36] and Qais and Ahmed [37] who evaluated the potential of several medicinal plants to check the quorum sensing and biofilm formation properties of disease-causing microorganisms.

### 3.5. Antibiofilm Activity

The antibiofilm activity of the ethanol extract of *A. aegerita* was carried out by the microtiter plate method and results are delineated in Table 4. With increasing concentration, the mushroom extract showed inhibition in biofilm formation in a concentration-dependent manner with a higher percentage of inhibition (72.35%) at 1000 µg/mL and the least percentage of inhibition (22.34%) at 125 µg/mL of concentration. In addition, the control sample showed significantly higher antibiofilm activity as compared to mushroom extract. The potential of mushroom extract to inhibit biofilms is due to the availability of phenolic compounds, namely gallic acid and rutin. The present results are in accordance with the results of Nassima et al. [38] who evaluated the potential of phenolic compounds isolated from extract prepared from *Populus nigra* and *Populus alba* bud.

## 4. Conclusions

The main objective of the study was to study the promising bioactive properties of ethanol extract obtained from *A. aegerita* mycelial culture prepared by a modified evaporation technique. The research highlighted that the characterization of the extract by FTIR showed the presence of functional groups of phenolic compounds, and quantification of the extract by HPLC analysis revealed the amount of gallic acid is significantly (*p* < 0.05) high in comparison to rutin. Furthermore, the extract exhibits remarkable antioxidant and antibiofilm activity, and effectively inhibits the virulence factors such as pyocyanin, pyoverdine, and swarming motility responsible for quorum sensing. Therefore, the present finding highlights the potential of phenolic compounds isolated from edible mushrooms and the possibility of developing new therapeutic agents from these mushrooms to combat drug-resistant pathogens. Further research is required to explore the therapeutic potential of the mushrooms and to optimize their utilization, thereby providing valuable insight for future advancements in healthcare, biotechnological, and food applications.

## Figures and Tables

**Table 1:** Identification of compounds present in ethanol extract of mycelial culture of *A. aegerita* mushroom.

Retention Time (min)	Compound Name	Molecular Formula
10.42	5-Methyl-1-heptanol	C_8_H_18_O
14.11	4-Nonene, 3-methyl-, (Z)-	C_10_H_20_
17.04	1-Hexadecanol	C_16_H_34_O
19.56	2-Heptadecenal	C_17_H_32_O
21.80	10-Heneicosene (c,t)	C_21_H_42_
23.66	Phthalic acid, butyl hept-4-yl ester	C_19_H_28_O_4_
25.69	2-Dodecanol	C_12_H_26_O
28.56	9H-Carbazole-1-carboxylic acid, 4-(1H-indol-3-yl)-, methyl ester	C_22_H_16_N_2_O_2_
30.38	Ethanol, 2-(methylamino)-	C_3_H_9_NO
33.57	Benzoic acid, TMS derivative	C_10_H_14_O_2_Si
36.73	Acetamide, 2,2,2-trifluoro-	C_2_H_2_F_3_NO
40.57	7-Hydroxy-7,8,9,10-tetramethyl-7, 8-dihydrocyclohepta[d,e]naphthale	C_18_H_20_O
42.55	Pyrazolo[1,5-a]pyridine, 3-methyl-2-phenyl	C_14_H_12_N_2_

**Table 2:** Antioxidant activity of ethanol extract of *A. aegerita*.

Concentration of Mushroom Extract (µg/mL)	DPPH Scavenging Assay (%)	Control Ascorbic Acid (%)	Nitric Oxide Scavenging Assay (%)	Control Ascorbic Acid (%)
50	38.53 ± 0.11 ^a^	42.33 ± 0.06 ^a^	38.82 ± 0.04 ^a^	43.47 ± 0.09 ^a^
100	54.31 ± 0.12 ^b^	57.43 ± 0.05 ^b^	51.63 ± 0.11 ^b^	53.67 ± 0.12 ^b^
150	72.83 ± 0.11 ^c^	78.60 ± 0.11 ^c^	69.91 ± 0.69 ^c^	68.37 ± 0.17 ^c^
200	85.63 ± 0.12 ^d^	95.90 ± 0.21 ^d^	82.02 ± 0.12 ^d^	83.34 ± 0.35 ^d^

The results were expressed as mean ± standard deviation of >3 independent replicates, while different lowercase letters (a–d) in the same row represent significant differences (*p* < 0.05) from each other.

**Table 3:** Effect of ethanol extract of *A. aegerita* on virulence factor of *P. aeruginosa* PAO1.

Mushroom Extract (µg/mL)	Pyocyanin (µg/mL)	Pyoverdine (RFU)	Swarming Motility (mm)
200	14.43 ± 0.09 (11.68%)	754.92 ± 0.17 (10.78%)	17.37 ± 0.09 (11.3%)
400	12.34 ± 0.12 (24.47%)	634.51 ± 0.24 (25.01%)	14.36 ±0.12 (26.7%)
600	10.87 ± 0.05 (33.47%)	556.82 ± 0.15 (34.19%)	12.54 ± 0.05 (36.02%)
800	8.24 ± 0.07 (49.57%)	468.31 ± 0.12 (44.65%)	10.82 ± 0.07 (44.7%)
1000	6.32 ± 0.12 (61.32%)	389.02 ± 0.015 (54.02%)	8.56 ± 0.06 (56.32%)
Control	16.34 ± 0.10	846.21 ± 0.18	19.62 ± 0.05

**Table 4:** Antibiofilm activity of ethanol extract of *A. aegerita* against *P. aeruginosa* PAO1.

Concentration of Mushroom Extract (µg/mL)	Biofilm Inhibition Extract (%)	Biofilm Inhibition Streptomycin (Control) (%)
200	22.34 ± 0.21 ^a^	36.45 ± 0.56 ^b^
400	32.67 ± 0.17 ^a^	47.34 ± 0.32 ^b^
600	49.48 ± 0.22 ^a^	58.67 ± 0.26 ^b^
800	61.24 ± 0.28 ^a^	69.56 ± 0.19 ^b^
1000	72.35 ± 0.14 ^a^	84.23 ± 0.18 ^b^

The results were expressed as mean ± standard deviation of >3 independent replicates; different lowercase letters (a,b) in each row represent significant differences (*p* < 0.05) from each other.

## Data Availability

The data used to support the findings of this study can be made available by the corresponding author upon request.
